# A wearable ring-shaped inertial system to identify action planning impairments during reach-to-grasp sequences: a pilot study

**DOI:** 10.1186/s12984-021-00913-4

**Published:** 2021-07-27

**Authors:** Erika Rovini, Guenda Galperti, Valeria Manera, Gianmaria Mancioppi, Laura Fiorini, Auriane Gros, Philippe Robert, Filippo Cavallo

**Affiliations:** 1 Department of Industrial Engineering, University of Florence, Florence, Italy; 2 The BioRobotics Institute of Scuola Superiore Sant’Anna, Pontedera, Pisa, Italy; 3 Department of Excellence in Robotics & AI, Scuola Superiore Sant’Anna, Pisa, Italy; 4 CoBTeK Lab of the Université Cote D’Azur, Nice, France

**Keywords:** Action planning, Dysexecutive syndrome, Executive functions, Mild cognitive impairment, Motor programming, Reach-to-grasp

## Abstract

**Background:**

The progressive ageing of the population is leading to an increasing number of people affected by cognitive decline, including disorders in executive functions (EFs), such as action planning. Current procedures to evaluate cognitive decline are based on neuropsychological tests, but novel methods and approaches start to be investigated. Reach-to-grasp (RG) protocols have shown that intentions can influence the EFs of action planning. In this work, we proposed a novel ring-shaped wearable inertial device, SensRing, to measure kinematic parameters during RG and after-grasp (AG) tasks with different end-goals. The aim is to evaluate whether SensRing can characterize the motor performances of people affected by Mild Neurocognitive Disorder (MND) with impairment in EFs.

**Methods:**

Eight Individuals with dysexecutive MND, named d-MND, were compared to ten older healthy subjects (HC). They were asked to reach and grasp a can with three different intentions: to drink (DRINK), to place it on a target (PLACE), or to pass it to a partner (PASS). Twenty-one kinematic parameters were extracted from SensRing inertial data.

**Results:**

Seven parameters resulted able to differentiate between HC and d-MND in the RG phase, and 8 features resulted significant in the AG phase. d-MND, indeed, had longer reaction times (in RG PLACE), slower peak velocities (in RG PLACE and PASS, in AG DRINK and PLACE), longer deceleration phases (in all RG and AG DRINK), and higher variability (in RG PLACE, in AG DRINK and PASS). Furthermore, d-MND showed no significant differences among conditions, suggesting that impairments in EFs influence their capabilities in modulating the action planning based on the end-goal.

**Conclusions:**

Based on this explorative study, the system might have the potential for objectifying the clinical assessment of people affected by d-MND by administering an easy motor test. Although these preliminary results have to be investigated in-depth in a larger sample, the portability, wearability, accuracy, and ease-of use of the system make the SensRing potentially appliable for remote applications at home, including analysis of protocols for neuromotor rehabilitation in patients affected by MND.

## Introduction

The progressive ageing of the global population is leading to an increasing number of people affected by cognitive decline and dementia [1]. Particularly, it is expected that the number of people suffering from Alzheimer’s Disease (AD) (accounting for 60–65% of the dementia cases) will reach 74.7 million by 2030 and 100 million by 2050 [2]. Even though dementia is mainly associated to the prototypic memory loss, different cognitive domains can be affected by different pathologies, leading to distinct cognitive symptoms. Among them, the executive functions (EFs) represent a complex construct that involves cognitive, behavioural, and emotional aspects. Deficits in EFs can be defined as “*dysexecutive syndrome*” [3], which includes cognitive (e.g., deficits in response inhibition, rules deduction, set-shifting, information generation, action planning, response initiation, coordination of dual-tasks) and/or behavioural (e.g., hypoactivity, apathy, distractibility, preservative behaviour, social behaviour) alterations [4].

Currently, EFs are clinically evaluated mainly administering standardized neuropsychological tests [5] such as the Frontal Assessment Battery (FAB) and the Behavioral Assessment of Dysexecutive Syndrome (that evaluate the EFs as a whole), and tests assessing specific aspects of EFs, such as the Trail Making Test (TMT, for divided attention and working memory), the Stroop Interference Test (for response inhibition), the Digit Span (for verbal working memory), or the Tower of London (for planning).. Although neuropsychological testing is today the gold standard to assess dysexecutive symptoms, a recent literature review highlighted that they present several limitations. The validity and reliability of the test results are sometimes limited because of normative data based on small datasets, some of the cognitive domains are scarcely represented, while others are assessed in different tasks, many tests are available in a restricted number of languages, and sometimes cultural habits can affect the execution of the required tasks [6].

In this context, new protocols and novel tools to assess neuropsychological functions should be investigated. In this work, we focus on the decline of EFs in motor programming that results in action planning impairments.

In the past years, some experimental studies have proposed reach-to-grasp (RG) protocols to highlight how the intentions can influence the action planning. RG sequences analyses have revealed that healthy subjects differently reach and grasp an object depending on the action final goal [7] because people are driven by prior intentions. Kinematics conveys information about these intentions [8], so that, even if the object to-be-grasped is the same, different motor parameters can be appreciated [9]. Therefore, when someone reaches and grasp a bottle to pour its content into a container or, conversely, to pass it to someone else, modulation of the kinematic action occurs. Furthermore, previous studies that implemented experimental protocols based on RG and after-grasp (AG) sequences have revealed useful information in several pathologies, such as Parkinson’s Disease [9, 10], autism spectrum disorder [11, 12], and stroke [13].

Reach-to-grasp tests are easy to be performed and can overcome languages and cultural bias. Nevertheless, up to now, the traditional methodologies employed to analyze motor performance during such tasks are mainly based on motion optical capture systems, which are expensive, require lengthy procedures and dedicated staff for set-up and analysis, and are applicable in dedicated wide settings only.

Recently, advances in Micro Electro-Mechanical Systems, and in artificial intelligence (AI) techniques, have allowed employing wearable technology, together with processing and learning algorithms, to evaluate experimental protocols, both in lab and in clinical settings. This represents a promising solution for objective and reliable monitoring, assessment, and support [14]. Hence, wearable inertial devices have been used, so far, to acquire and process high-frequency rate data to analyze motion performances within several applications, including daily activity living gestures [15], early Alzheimer’s detection [16], Parkinson’s disease assessment [17], mild cognitive impairment evaluation [18], and autism spectrum disorder assistance [19].

In this context, we propose SensRing: a non-invasive, low-cost, lightweight, easy-to-use, ergonomic device able to capture the 3D movements of a finger in the space. In a previous work, we evaluated the accuracy of this device in measuring kinematic parameters in healthy people during standard exercises [20]. Here, the device is proposed within a pilot study, for the use in a clinical application with people suffering from mild cognitive decline and healthy controls. The SensRing allows the measurement of the kinematic parameters related to the motor performance without interfering on motion capabilities.

This study proposes the use of SensRing as an alternative approach to traditional methods aiming to objectively analyze RG and AG sequences. Since Mild Neurocognitive Disorder (MND) subjects, with a decline in EFs, often show impairment in motor programming and action planning [21], the cognitive decline could be objectively confirmed through kinematic parameters’ variations. The hypothesis is that the cognitive decline could be identified objectively examining the kinematic parameters. The idea is to investigate: (i) whether the kinematic performance of people diagnosed as MND with EF impairment is different compared to older healthy controls, during a simple motor protocol, and (ii) whether there are differences in action kinematic modulation depending on the action end-goal between MND and healthy subjects. Finally, we evaluated whether the kinematic parameters could be correlated to the traditional cognitive assessment based on clinical scores.

## Materials and methods

### Participants

Ten healthy controls (HC) (6 females, 4 males, mean age ± standard deviation (SD) 63.7 ± 9.9 years old) and 17 subjects diagnosed as Mild Neurocognitive Disorder (MND) (13 females, 4 male, mean age ± SD 77.1 ± 5.15 years old) were recruited at the Nice Research Memory Center (CMRR) & Cognition Behaviour Technology laboratory (CoBTeK). All the subjects were recruited in the context of Marco-Sense multi-centric research protocol, which was performed in accordance with the Declaration of Helsinki and approved by the ethical committee CPP Ile de France (N° IDRCB: 2019-A00342-55). All participants received detailed written explanations on the study and signed written informed consent. Participants were not included in the study if they had a score at the Mini-Mental State Examination (MMSE) < 22 [22] and a Frontal Assessment Battery (FAB) score < 11 [23]. All the participants were right-handed. Out of the 17 MND subjects, 8 subjects (7 females, 1 male, mean age ± SD 75.7 ± 5.5 years old) presented dysexecutive MND impairments (e.g., action planning and motor programming deficits), and as individuals with dysexecutive MND (d-MND) were involved in this study. The presence of dysexecutive deficits was based on the official DSM-5 diagnosis reported in the clinical records of Bank National Alzheimer. Diagnoses reported in the BNA are performed by expert clinicians based on the patients’ clinical, behavioral and neuropsychological profiles; all patients included in the study were already in the CMRR database.

### Instruments

**Fig. 1:**
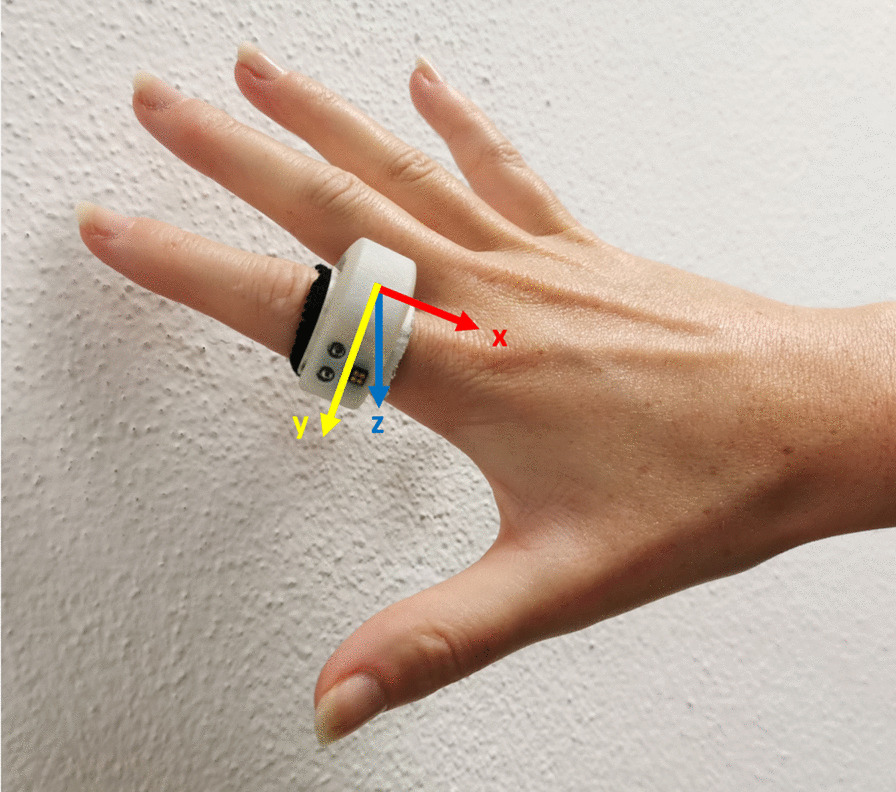
SensRing wearable device. The direction and orientation of sensor axes are reported (x‐axis in red, y‐axis in yellow, z‐axis in blue)

A novel ring-shaped device, called SensRing (Fig. 1), has been developed at the BioRobotics Institute of Scuola Superiore Sant’Anna (Pisa, Italy) to fully track the orientation and movement of the finger where it is worn [20]. The device, based on an ARM®Cortex™-M3 32-bit STM32-F103 microcontroller (STMicroelectronics, Italy), can acquire and store data with 50 Hz sampling frequency. SensRing mounts a 9–axes inertial measurement unit (IMU) LSM9DS1 (STMicroelectronics, Italy), including a 3D digital linear acceleration sensor (selectable full scale: ± 2/ ± 4/ ± 8/ ± 16 g), 3D digital angular rate sensor (selectable full scale: ± 245/ ± 500/ ± 2000 dps), and 3D digital magnetic sensor (selectable full scale: ± 4/ ± 8/ ± 12/ ± 16 gauss). SensRing selected 2 g, 2000 dps, and 4 gauss as full scales. Default values for calibration were used. Data from magnetometer were acquired but not used in this study. An integrated Bluetooth module (Rigado BMD-350, Nordic Semiconductor, Norway) allows wireless communication for data transmission towards a generic control station. A dedicated interface, developed in Visual Studio 2019 (Microsoft Corporation, USA) and based on C# language, ensures managing the connection and the acquisition of sensors data. A small, rechargeable PoLi battery, externally fixed to the wrist with an elastic band, supplies the SensRing. Integrated solutions for the battery are currently under development. For this study, an elastic band was fixed on the plastic ring to make it adaptable to different fingers sizes (as visible in Fig. 1). The elastic band ensures easy wearability of the device, without inferring with the required movements.

### Experimental protocol

**Fig. 2:**
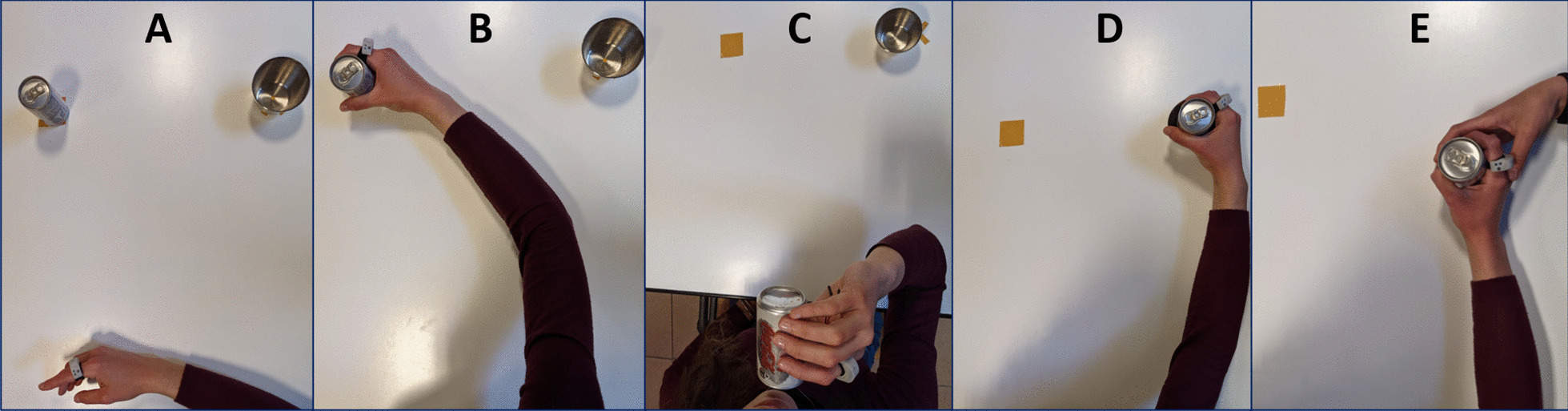
The experimental conditions: in** A**, the starting position for all the tasks; in** B**, the grasping moment for all the tasks; in** C**, the final position for the drinking action (DRINK); in** D**, the final position of the placing action (PLACE); in** E**, the final position of the passing action (PASS)

Before starting the trial, the experimenters required the subjects to wear SensRing on the proximal phalanx of the dominant index finger. Participants sat in front of a table, laying their dominant hand on the starting position (3 cm from the edge of the table midsagittal position, and 15 cm away from the midsection). Experimenters instructed the participants to perform shorts reach-to-grasp (RG) and after-grasp (AG) sequences with three different end-goals, adapted from a previous study [7]. For each task, a can (ø = 5 cm, h = 8.5 cm) has been positioned in front of the participant, at 21 cm from the hand starting position along the midsagittal plane. The initial position was acquired as a baseline for 5 s. Afterwards, a tone indicated the beginning of the task. For each condition, 10 repetitions were performed. During the drinking condition (DRINK), subjects had to reach the can, grasp it, and lift it up simulating a drinking action. On the other hand, during the placing condition (PLACE), subjects had to reach the can, grasp it and place it inside a cup (ø = 7 cm), located 28 cm at the right side with respect to the initial position of the can. Finally, the passing condition (PASS) required the subjects to reach the can, grasp it and pass it to a partner. The partner sat to the right side of the table with the hand resting (on the same position of the cup in the previous condition) ready to take the can. Both for PLACE and PASS, the can was repositioned on the initial position after each repetition. The order of conditions was randomized across participants. The entire protocol lasted about 10 min, considering the wearing of SensRing, the explanation for correctly carrying out the test, and the test execution. A graphical representation of the experimental protocol is reported in Fig. 2.

### Signal processing and feature extraction

Inertial data acquired with SensRing were stored and offline processed by using MATLAB R2018a (The MathWorks, Inc., Natick, MA, USA). Accelerations and angular velocities, acquired from the accelerometer and gyroscope integrated into the IMU, were pre-processed with a fourth-order low-pass digital Butterworth filter, using a 5 Hz cut-off frequency to delete high-frequency noise.

**Fig. 3:**
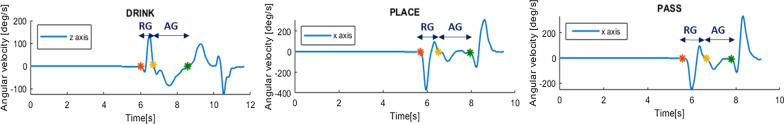
Signal segmentation to extract characteristic phases; RG phase from the starting time (in red) to the grasping time (in yellow), and AG phase from the grasping time to the end time (in green)

Two characteristic phases were identified in each repetition: the RG phase from the beginning of the action to the grasping of the object, and the AG phase from the object grasping to the end of the task. Accordingly, custom algorithms were implemented to segment the signal into these phases, across each exercise. Preliminary analysis carried out with the Vicon optoelectronic system and the SensRing allowed us to correctly segment the signal through the use of x,y,z, coordinates of the Vicon’s markers attached in specific parts of the hand that let us better identify the different phases of the task. Particulary, the dominant axis of the angular velocity was used as the reference signal for the segmentation, and three characteristic times were calculated for each repetition: the starting time, the grasping time, and the end time (Fig. 3). To select the dominant axis, we preliminary visually checked the main direction of the movement, plotting the velocity along the three axes. Then, a set of kinematic parameters was extracted from accelerations and angular velocities as detailed in Table 1, to investigate different aspects of the motor performances including, for instance, energy, duration, velocity [24]–[26]. All the parameters were computed offline. For some parameters, such as peak velocities and index finger excursions, a linear drift correction was applied to remove the offset after the integration.

**Table 1:** Kinematic parameters extracted from SensRing

Parameter	Meaning	Phase
*IAV*	Integral of the magnitude of the acceleration vector: it represents a value correlated to the energy expenditure (m/s)	RG, AG
*Vpeak*	Maximum value of the amplitude of the peak velocity (m/s) calculated from the integration of the magnitude acceleration vector	RG, AG
*T_Vpeak*	Time of peak velocity: it is the time instant corresponding to the peak velocity (s)	RG, AG
*T_decel*	Deceleration time: it is the duration of the deceleration phase (s)	RG, AG
*Decel%*	Deceleration time (%): it represents the percentage of the deceleration phase compared to the total execution time	RG, AG
*T_exec*	Execution time: it represents the time spent to perform the movement (s)	RG, AG
*T_react*	Reaction time: it is the time elapsed from the start acoustic input to the beginning of the movement (s)	RG
*Index_exc*	Amplitude of maximum index finger excursion: it indicates the angular excursion of the index finger during the grasping of the object (deg). It is a measure related to the hand opening but estimated by using the index finger	RG
*T_index_exc*	Time of maximum index finger excursion: it is the time instant corresponding to the maximum excursion of the index finger during the grasping action (s)	RG
*Skew*	Skewness: it is a measure of the asymmetry of the distribution. It is calculated from the magnitude of the acceleration vector	RG, AG
*Kurt*	Kurtosis: it is a measure of the shape of the tail of the distribution. It is calculated from the magnitude of the acceleration vector	RG, AG
*rmseJerk*	Root mean square of the jerk, that is the rate of change of the acceleration: it represents the smoothness of the movement (m/s^3^)	RG, AG

*RG*  Reach-to-Grasp, *AG* After-Grasp

All the parameters were measured for each repetition both in the RG (i.e., from the starting time to the grasping time, see Fig. 3) and the AG (i.e., from the grasping time to the end time, see Fig. 3) phases, except for the reaction time, the amplitude and time of maximum index finger excursion that were calculated during the RG phase only. Totally, 21 parameters composed the dataset of each exercise (i.e., 12 features for the RG and 9 for the AG phase) as detailed in Table 1. The average value over the 10 repetitions was calculated and reported. The processing time required to extract all the features from each action over the 10 repetitions is about 3 s, estimated by using the function *tic-toc* of Matlab R2018a.

### Data analysis

Qualitative variables, such as gender and education level, were compared using Chi2 test, whereas the remaining clinical data measured, as quantitative variables, through the Mann–Whitney U-test. The Kolmogorov–Smirnov test was preliminarily applied to verify the data distribution of each extracted parameter (see Table 1). Since all parameters resulted as not normally distributed, non-parametric statistical tests were adopted for data analysis.

Specifically, two macro-analyses were carried out:

1. Inter-group analysis: to evaluate if kinematic parameters may be able to differentiate HC and d-MND in terms of action planning and performance. The non-parametric Mann–Whitney U-test was applied to investigate significant differences (p < 0.05) between the two groups. Also, the effect size was measured by calculating the Cohen's d [27] to further investigate the differences between groups of those parameters that showed significant p-value.
2. Intra-group analysis: to investigate, within each group, if motor patterns are modulated based on the action planning and the execution of three conditions with different end-goal. The Friedman test was applied to test the three experimental conditions. Then, non-parametric Wilcoxon post-hoc analysis was used with Bonferroni correction for correcting multiple testing.

Additionally, a correlation analysis of the motor performance to the MMSE score was executed for each parameter, calculating the Spearman’s correlation coefficients. This analysis investigates whether the motor parameters correlate to the clinical score of a standard neuropsychological test [28], typically used as a screening tool for cognitive assessment. d-MND and HC are considered as a unique group for this analysis.

## Results

Subjects’ demographic and clinical characteristics were reported in Table 2. A significant statistical difference on age has been attested between the two groups. Conversely, no differences emerged regarding the educational level and the gender of participants. Moreover, as expected, the two groups differed in terms of the global level of cognitive functioning, according to the MMSE score (p < 0.0001).

**Table 2:** Clinical and Demographical data

	HC (10)	d-MND (8)	p-value
Female (%)	60.0%	87.5%	0.1955
Age (years)	63.7 ± 9.9	75.7 ± 5.2	0.0283
Education (%)			
Primary	0 (0.0%)	0 (0.0%)	0.3871
Secondary	3 (30.0%)	4 (50.0%)	
Higher	7 (70.0%)	4 (50.0%)	
MMSE	30.00 ± 0.00	24.25 ± 2.87	< 0.0001

*MMSE* Mini Mental State Examination score, *HC*  Healthy Controls; *d-MND* Individuals with dysexecutive MND

### Intergroup analysis

Healthy subjects differed from d-MND in some kinematic parameters. Summarizing, 7 parameters differentiated the two groups in the RG phase, while 8 were significant in the AG phase. Results from the RG phase are reported in Table 3 for all the conditions (DRINK, PLACE, or PASS), whereas complete results for the AG phase are shown in Table 4.

**Table 3:** Mean values and standard deviation (SD) of significant kinematic parameters for HC And d-MND at inter-group analysis during reach-to-grasp (RG) phase

*Parameter*	DRINK		PLACE		PASS	
	HC	d-MND	HC	d-MND	HC	d-MND
*IAV*	8.49 ± 1.61	9.55 ± 1.97	9.64 ± 1.52	10.65 ± 0.98	10.13 ± 1.43	10.88 ± 1.20
*rmseJerk*	10.58 ± 6.04	8.77 ± 3.45	9.77 ± 2.78	9.88 ± 3.80	9.28 ± 3.54	8.45 ± 2.74
*Skew*	0.38 ± 0.08	0.41 ± 0.13	0.29 ± 0.05	0.42 ± 0.13*	0.43 ± 0.11	0.37 ± 0.18
*Kurt*	2.29 ± 0.13	2.52 ± 0.44	2.56 ± 0.25	2.76 ± 0.27	2.67 ± 0.20	2.83 ± 0.45
*Vpeak*	1.17 ± 0.54	0.78 ± 0.32	1.33 ± 0.38	1.02 ± 0.35 *	1.13 ± 0.22	0.84 ± 0.28 *
*T_Vpeak*	0.36 ± 0.04	0.35 ± 0.03	0.36 ± 0.05	0.35 ± 0.05	0.35 ± 0.03	0.34 ± 0.05
*T_decel*	0.50 ± 0.14	0.70 ± 0.27	0.62 ± 0.13	0.75 ± 0.10	0.61 ± 0.16	0.76 ± 0.11*
*T_decel_perc*	56.64 ± 4.89	63.58 ± 8.01*	62.42 ± 4.21	68.32 ± 4.48*	65.54 ± 2.86	68.18 ± 6.20
*T_exec*	0.88 ± 0.16	0.99 ± 0.20	0.99 ± 0.15	1.10 ± 0.10	1.04 ± 0.14	1.13 ± 0.12
*T_react*	0.71 ± 0.11	0.77 ± 0.10	0.67 ± 0.10	0.81 ± 0.14*	0.67 ± 0.10	0.77 ± 0.10
*Index_exc*	65.39 ± 15.83	55.99 ± 10.27	60.85 ± 11.36	51.41 ± 6.32	61.77 ± 18.54	56.77 ± 9.48
*T_index_exc*	0.69 ± 0.14	0.74 ± 0.10	0.63 ± 0.11	0.68 ± 0.06	0.68 ± 0.19	0.69 ± 0.07

* Significant difference between healthy controls (HC) and dysexecutive MND subjects (d-MND) for each condition (i.e., Drink, Place and Pass actions); the * is reported in the d-MND column (p < 0.05)

**Table 4:** Mean values and standard deviation (SD) of significant kinematic parameters for HC and d-MND at inter-group analysis during after-grasp (AG) phase

Parameter	Drink		Place		Pass	
	HC	d-MND	HC	d-MND	HC	d-MND
*IAV*	14.56 ± 5.98	19.61 ± 9.08	13.18 ± 2.58	14.70 ± 3.85	12.39 ± 3.54	15.00 ± 5.91
*rmseJerk*	8.62 ± 4.39	5.29 ± 2.84*	6.08 ± 1.89	5.38 ± 1.38	4.36 ± 1.83	3.64 ± 0.68
*Skew*	0.34 ± 0.18	0.57 ± 0.24*	0.35 ± 0.14	0.39 ± 0.12	0.23 ± 0.08	0.45 ± 0.24*
*Kurt*	2.22 ± 0.40	2.94 ± 1.01	2.57 ± 0.31	3.01 ± 0.55	2.07 ± 0.23	2.68 ± 0.38*
*Vpeak*	2.92 ± 0.94	1.73 ± 0.81*	1.26 ± 0.20	0.95 ± 0.32*	0.73 ± 0.23	0.60 ± 0.16
*T_Vpeak*	0.63 ± 0.13	0.68 ± 0.20	0.35 ± 0.07	0.32 ± 0.07	0.32 ± 0.08	0.31 ± 0.08
*T_decel*	0.75 ± 0.35	1.28 ± 0.65*	0.99 ± 0.21	1.18 ± 0.30	0.88 ± 0.19	1.13 ± 0.44
*T_decel_perc*	50.49 ± 6.36	63.01 ± 0.79*	73.69 ± 4.14	77.31 ± 2.77	73.03 ± 10.02	74.58 ± 5.62
*T_exec*	1.51 ± 0.64	2.04 ± 0.94	1.36 ± 0.27	1.53 ± 0.39	1.27 ± 0.27	1.55 ± 0.61

*Significant difference between healthy controls (HC) and dysexecutive MND (d-MND) for each condition (i.e., Drink, Place and Pass actions); the * is reported into the d-MND column (p<0.05)

Trends characterizing the motor performance of the two groups were recognizable. For RG, HC reached a higher peak velocity compared to d-MND performing faster movements in PLACE and PASS conditions (Table 3). Despite longer execution time observed in d-MND group, no significant differences were detected in execution time in any conditions. The reaction times in d-MND were longer in PLACE condition. Moreover, d-MND subjects showed a longer deceleration phase in PASS, but the relative value with respect to the entire execution time of the action (T_decel_perc) was significant in DRINK and PLACE conditions. Additionally, the higher skewness in d-MND in PLACE, confirmed the wider variability in performing repetitive tasks in patients with respect to HC.

Even if the RG phase is the most significant to investigate impairments in action planning, similar trends were found also in the AG phase for RMSE_Jerk (DRINK), skewness (DRINK and PASS), kurtosis (PASS), Vpeak (DRINK and PLACE), T_decel (DRINK), and T_decel_perc (DRINK) (see Table 4), enhancing the potentiality of these parameters in identifying motor impairments in patients affected by the dysexecutive syndrome.

To further investigate the differences between the two groups in this exploratory analysis, the effect sizes (Cohen's d) was calculated for those parameters able to differentiate the two groups. Results are reported in Table 5. All the investigated parameters showed relevant effect size (i.e., effect size higher than 0.2 according to Coehn [27], ranging from small [d = 0.20–0.49] to medium [d = 0.50–0.79] effect.

**Table 5:** Effect size (Cohen’s d) of Significant parameters to distinguish between healthy controls and dysexecutive MND

Parameter	Phase	Drink	Place	Pass
*rmseJerk*	AG	0.379	–	–
*Skew*	RG	–	− 0.436	–
*Skew*	AG	− 0.418	–	− 0.412
*Kurt*	AG	–	–	− 0.680
*Vpeak*	RG	–	0.385	0.468
*Vpeak*	AG	0.557	0.459	–
*T_decel*	RG	–	–	− 0.366
*T_decel*	AG	0.384	–	–
*T_decel_perc*	RG	− 0.427	− 0.447	–
*T_react*	RG	–	− 0.432	–

*RG* reach-to-grasp phase, *AG* after grasp phase

### Intragroup analysis

Comparing the three tasks with different end-goals within each group, several significant differences came out. It is important investigating this comparison in the RG phase, which was the same for all the tasks.

**Fig. 4:**
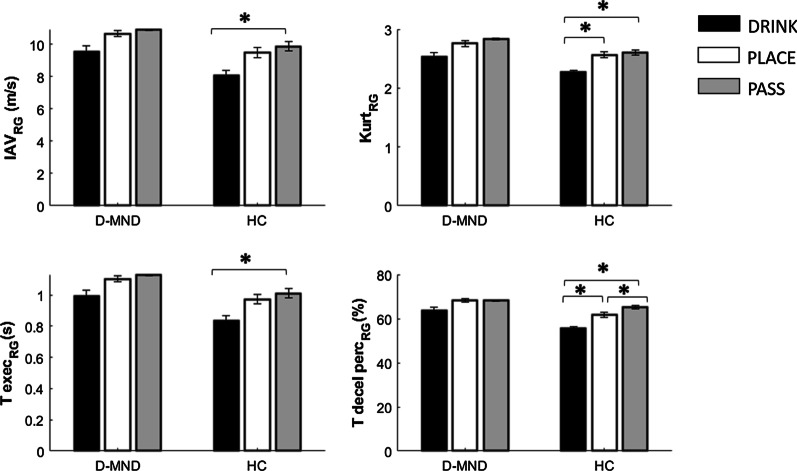
Significant results (*p < 0.05) at intragroup analysis during RG phase

As expected, HC showed differences in RG among conditions, whereas no significant differences were found in d-MND. Healthy subjects showed 7 parameters able to differentiate among tasks, showing that they modulated their kinematics according to the different action end-goal (Fig. 4) while d-MND did not show this capability.

IAV, kurtosis, and T_exec parameters had an increasing trend in values from DRINK, through PLACE, to PASS, both for d-MND and for HC. The passing condition, indeed, seemed to be the most demanding task, showing the highest IAV value, accompanied by the highest kurtosis, the longest execution time, and the longest deceleration rate phase. Conversely, drinking appears the simplest task, requiring less efforts and shorter times. Furthermore, DRINK had a deceleration phase (T_decel_perc) lower than the other two conditions for both groups.

Notably, the different end-goal seemed not to influence the kinematic of the index finger excursion, neither the time when the hand reaches the maximum excursion approaching the can.

### Motor parameters correlation to clinical score

Analyzing the correlation between the measured parameters and clinical score assigned to each participant (both d-MND and HC) according to the MMSE, we found some relationships. In Table 6, the parameters that showed at least moderate correlation (i.e., rho ≥ 0.3 or rho ≤ − 0.3) according to Ratner [28] and a significant p-value (p < 0.05) were reported. Higher peak velocity (in DRINK and PASS), and lower kurtosis (in PLACE and PASS), skewness (in DRINK and PLACE) and deceleration time (in DRINK) were correlated with better scores of the MMSE. Practically, the more people were cognitively healthy intact, the faster (and decelerate for less time) and less variable the movements they made.

**Table 6:** Correlation analysis to MMSE score

Condition	Parameter	Phase	Rho	p-value
Drink	Skew	AG	− 0.612	0.007
Drink	Vpeak	AG	0.585	0.011
Drink	T_decel	AG	− 0.477	0.045
Place	Skew	RG	− 0.522	0.026
Place	Kurt	AG	− 0.491	0.039
Pass	Kurt	AG	− 0.636	0.005
Pass	Vpeak	RG	0.492	0.038

*RG* reach-to-grasp phase, *AG* after grasp phase, *Rho* correlation coefficient from Spearman’s correlation analysis

## Discussion

This work presents a pilot study, where SensRing, a novel wearable ring-shaped device, is proposed to analyse how motor performances vary during sequences of reach-to-grasp and after-grasp actions with different end-goals in healthy people and subjects with a mild decline in executive functions. Then, we investigate how these variations in motor performances can correlate with the cognitive decline. The accuracy of SensRing in measuring motor parameters in healthy subjects while performing standard tasks compared to a gold standard optoelectronic system has been already presented in [20]. In this work, we propose to use SensRing as an alternative, easy-to-use, small, wearable, non-invasive solution for clinical application in people affected by MND with deficits in EFs. Such a system, indeed, has the potential for objectifying the patients’ evaluation accurately, measuring their motor performances during a simple motor protocol. The choice of developing a ring instead of a watch is more novel and challenging. SensRing is lighter, more compact, and less invasive than a watch. Furthermore, in our study, we take into account also the grasping movement, and it is not possible to estimate this action by using a wrist-worn device. Additionally, wrist-worn devices cannot measure the fine movement of the finger, which is very important when evaluating motor decline caused by neurodegenerative disease. For example, in Parkinson’s disease, the repetitive movement of the finger is assessed to evaluate bradykinesia [17], and similar tasks can be used also for investigating cognitive impairment within motor cognitive dual-task paradigms [29]. Also, studying the optimal placement of the sensors for gesture recognition applications, the contribution of the index finger is very important compared to the accuracy provided by the wrist-worn sensor in detecting different gestures [15].

In this study, short RG and AG sequences with different end-goals were proposed to investigate whether the different intention can influence the planning and the action both in a group of HC and d-MND subjects. The proposed experimental protocol is easy to be set-up and quick to be performed. Furthermore, it is not constrained to language or cultural issues because it is based on simple motor sequences. The possibility to objectify the assessment of the tasks, complementing the neuropsychological testing through finer, quantitative measures related to motion performance could be a groundbreaking achievement in the field of early diagnosis of dementia, able to significantly improve the characterization of MND subjects. To the best of our knowledge, this is the first study that employs a non-invasive wearable ring-shaped device to measure the kinematics of the movement during RG sequences, aiming to evaluate whether differences in motor performances can characterize people affected by MND with a specific impairment of EFs.

The analysis of the accelerations and the angular velocities acquired by SensRing allows calculating a set of 21 motor parameters (i.e., 12 in RG phase and 9 in AG phase) that objectively characterize the kinematic of the subjects' motor performance.

We observed interesting patterns in several parameters when d-MND and HC subjects have been compared. These results endorse our hypothesis that EFs impairments reflect on worsening in motor performances. d-MND, indeed, needs more time to initiate the movement. Additionally, they move slower, also presenting longer deceleration phases. This suggests that a higher effort was required to d-MND to perform the same tasks of HC considering basic motor tasks. Increase of movement time spent decelerating, indeed, is associated with an onward action, that requires a greater level of precision [30]. More gradual acceleration changes and longer deceleration phases are reflected also in the measure of jerk, which is the derivative of the acceleration and results slower in d-MND respect to HC. Differently, HC have lower IAV than d-MND, confirming they spend less energy to carry out the movement that results in a more optimal and ecological performance. Also, higher kurtosis and skewness values for d-MND demonstrate higher variability of pathological subjects in performing the movements. An exception to this pattern is the PASS condition for healthy that could show higher variability when approaching a partner, differently from d-MND that less modulate the kinematic of the movement according to the end-goal of the action. Finally, HC anticipate the maximum index finger excursion with respect to d-MND, supporting the theory that action planning in subjects with EFs disorders is compromised and they require more time to organize the grasping action, independently from the forthcoming action. Furthermore, trends found for RG phase are mainly confirmed also in AG phase, strengthening the choice of these parameters for characterizing the motor pattern of the involved subjects. Additionally, even if the AG phase is affected by other factors that change depending on the task (e.g., drink is kinematically different from place and pass; the target in “place” is a small cup, whereas in “pass” the target is the hands of another person), being the second step of an action plan, it may also reflect the effects of cognitive load as it represents the second phase of an action sequence.

These preliminary results suggest the potentiality of this device in developing a decision support tool for clinical assessment of d-MND people, providing accurate motor measurements of the subjects’ performance while carrying out a simple fast protocol. Enlarging the dataset, in future works, we aim at deeper investigating the validity and reliability of the measured parameters in discriminating the two groups of subjects. Since this is a pilot study with an exploratory analysis, it would be worthy in the next work, investigating again both parameters that already showed significant differences in this work (e.g., deceleration rate time, skewness, kurtosis, peak velocity) and parameters that have revealed a trend without reaching statistical differences. A larger sample size, indeed, can confirm the validity of such parameters.

Regarding the kinematic variations as response to the different end-goal of the tasks, as suggested by [30, 31], we have found that healthy subjects differently modulated the movements in the reach-to-grasp sequences according to different intentions. Conversely, people presenting EFs deficits did not show the same capability, resulting in no significant parameters able to distinguish among the three conditions (drink, place, and pass); thus they do not seem to adjust their movement based on the forthcoming action. However, both HC and d-MND confirmed that more precise tasks require more effort and execution time, according to [30, 32]. Also, the deceleration phase is longer when for onward actions have higher-precision requirements. Probably, drinking is a more automatic movement, compared to place the can on a specific narrow target or to pass it to another person. Therefore, the kinematic of the first action is quite different from the other two. Also, using a simulation of drinking and not really drinking might influence the kinematics, because the goal of the drinking task is different from normal drinking and this can be a limitation of the current work. However, HC compared to d-MND can differentiate their performance also between the placing and the passing actions, demonstrating a finer ability in modulating kinematic according to the specific task end-goal.

Finally, the correlation of some features to the clinical score of a standard neuropsychological test (i.e., MMSE) typically used as a screening tool for cognitive assessment, confirm the validity of these kinematic parameters in characterizing the cognitive decline (i.e., better movements are performed by people without cognitive decline).

The difference in age between the two groups is a limitation of this work. The ageing process, indeed, can affect motor performances during RG protocols, resulting in slower, longer, and show a prolonged approach phase to the object for older adults [33]. This movement protraction can be explained as an increment of time that older persons need to develop compensative strategies [34]. However, protocols which involve highly goal-directed tasks, such as our experimental protocol, seem to mitigate the differences between older adults and controls [35]. Nevertheless, future studies should involve a larger dataset, including age-matched groups to highlight differences related to the cognitive decline. Additionally, further investigations could be performed considering the combined execution of motor planning and memory tasks [36], exploiting the paradigm of the motor and cognitive dual-task to provide insights about the cognitive resources distribution in patients with dysexecutive syndrome.

## Conclusion

In this pilot study, SensRing has been employed to analyze the motor performances of healthy controls and subjects suffering from mild cognitive impaired, particularly a decline in executive functions, highlighted during a simple motor protocol based on reach-to-grasp and after-grasp sequences. The cognitively impaired subjects showed deficits in motor performance (e.g. slower movement, slower reaction, longer deceleration phases) compared to healthy subjects. Furthermore, they were not prone to modulate the kinematic of their actions during the reach-to-grasp phase (which is identical for all the tasks) when we compared similar tasks with different end-goal, evidencing impairments in action planning. Since this is a pilot study, we propose to further investigate in a larger sample the results obtained in this explorative study about the use of SensRing to objectify the clinical evaluation of people suffering from mild cognitive decline, implementing a simple motor test easy to set up. Analysing reach-to-grasp sequences, indeed, SensRing can measure kinematic parameters that have the potential to characterize the motor profile of patients with mild cognitive impairment. Such a system might have the potential to be use for clinical application, and then it could be also usable at home, for remote monitoring and eventually for analyzing specific tasks for neuromotor rehabilitation in patients affected by MND.

## Data Availability

The datasets used and/or analysed during the current study are available from the corresponding author on reasonable request.

## Abbreviations

DRINK: The experimental condition to drink
PASS: The condition to pass the can to a partner
PLACE: The condition to place the can on a target
EFs: Executive functions
d-MND: Individuals with dysexecutive MND, that are MND subjects with impairment in EFs
MND: Mild neurocognitive disorder
HC: Healthy controls
RG: Reach-to-grasp action
AG: After-grasp action
MMSE: Mini Mental Score Examination
